# Bone marrow stromal antigen 2 (BST-2) genetic variants influence expression levels and disease outcome in HIV-1 chronically infected patients

**DOI:** 10.1186/s12977-022-00588-2

**Published:** 2022-01-26

**Authors:** Hlelolwenkosi Mlimi, Kewreshini K. Naidoo, Jenniffer Mabuka, Thumbi Ndung’u, Paradise Madlala

**Affiliations:** 1 HIV Pathogenesis Programme, The Doris Duke Medical Research Institute, Nelson R. Mandela School of Medicine, University of KwaZulu-Natal, 719 Umbilo Road, Durban, 4013 South Africa; 2 School of Laboratory Medicine and Medical Sciences, University of KwaZulu-Natal, Durban, South Africa; 3 Africa Health Research Institute (AHRI), Durban, KwaZulu-Natal South Africa; 4 Ragon Institute of Massachusetts General Hospital, Massachusetts Institute of Technology, and Harvard University, Cambridge, MA 02139 USA; 5 Division of Infection and Immunity, University College London, London, UK; 6 Department of Global Health, University of Washington, Seattle, USA

**Keywords:** BST-2, SNPs, Expression levels, gp120- and p24-IgG levels, Viral loads

## Abstract

**Background:**

Bone marrow stromal antigen 2 (BST-2) also known as Tetherin (CD317/HM1.24), is a host restriction factor that blocks the release of HIV-1 virions from infected cells. Previous studies reported that BST-2 genetic variants or single nucleotide polymorphims (SNPs) have a preventative role during HIV-1 infection. However, the influence of BST-2 SNPs on expression levels remains unknown. In this study, we investigated the influence of BST-2 SNPs on expression levels and disease outcome in HIV-1 subtype C chronically infected antiretroviral therapy naïve individuals.

**Results:**

We quantified BST-2 mRNA levels in peripheral blood mononuclear cells (PBMCs), determined BST-2 protein expression on the surface of CD4^+^ T cells using flow cytometry and genotyped two intronic single nucleotide polymorphisms (SNPs) rs919267 and rs919266 together with one SNP rs9576 located in the 3’ untranslated region (UTR) of *bst-*2 gene using TaqMan assays from HIV-1 uninfected and infected participants. Subsequently, we determined the ability of plasma antibody levels to mediate antibody-dependent cellular phagocytosis (ADCP) using gp120 consensus C and p24 subtype B/C protein. Fc receptor-mediated NK cell degranulation was evaluated as a surrogate for ADCC activity using plasma from HIV-1 positive participants. BST-2 mRNA expression levels in PBMCs and protein levels on CD4^+^ T cells were lower in HIV-1 infected compared to uninfected participants (p = 0.075 and p < 0.001, respectively). rs919267CT (p = 0.042) and rs919267TT (p = 0.045) were associated with lower BST-2 mRNA expression levels compared to rs919267CC in HIV-1 uninfected participants. In HIV-1 infected participants, rs919267CT associated with lower CD4 counts, (p = 0.003), gp120-IgG1 (p = 0.040), gp120-IgG3 (p = 0.016) levels but higher viral loads (p = 0.001) while rs919267TT was associated with lower BST-2 mRNA levels (p = 0.046), CD4 counts (p = 0.001), gp120-IgG1 levels (p = 0.033) but higher plasma viral loads (p = 0.007). Conversely, rs9576CA was associated with higher BST-2 mRNA expression levels (p = 0.027), CD4 counts (p = 0.079), gp120-IgG1 (p = 0.009), gp120-IgG3 (p = 0.039) levels but with lower viral loads (p = 0.037).

**Conclusion:**

Our findings show that *bst-*2 SNPs mediate BST-2 expression and disease outcome, correlate with gp120-IgG1, gp120-IgG3 levels but not p24-IgG levels, ADCC and ADCP activity.

**Graphical Abstract:**

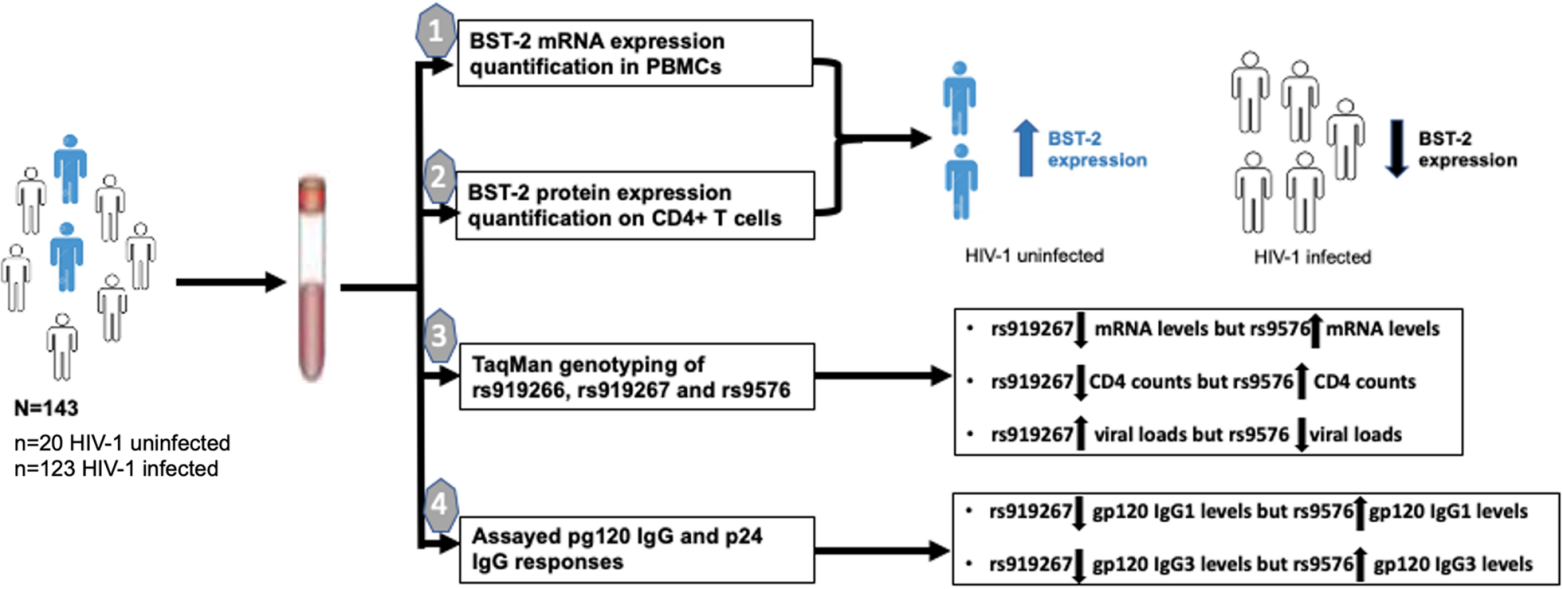

**Supplementary Information:**

The online version contains supplementary material available at 10.1186/s12977-022-00588-2.

## Background

Bone marrow stromal antigen 2 (BST-2; also known as CD317, and tetherin) is a host restriction factor that blocks the release of enveloped viruses, including HIV-1, from infected host cells [1] and modulates plasmacytoid dendritic cell (pDC) interferon (INF)-α production [2, 3]. BST-2 is probably a receptor that confers anti-viral antigen presentation properties to pDCs, given the high expression of BST-2 in pDCs following activation [4–6]. Although the tethering capacity of BST-2 was first described for HIV-1 [1], it was subsequently shown to occur for other enveloped viruses [7]. While BST-2 can be found in the trans‐Golgi network and in vesicular compartments, it is a transmembrane protein primarily located on the apical membrane (reviewed in [8]). The acute “murine AIDS’’ (LP-BM5) and/or Moloney murine leukaemia virus (Mo-MuLV) replication was not different between the wild type (WT) and BST-2 knockout (KO) mice [6, 9]. However, a different study reported that even though WT and BST-2 KO mice had similar acute LP-BM5 replication levels, BST-2 KO mice had higher replication levels during later time points, when adaptive immune responses operate [10]. Another study reported that BST-2 KO mice had higher acute mouse mammary tumour virus (MMTV) replication levels [11].

The BST-2 protein is encoded by *bst-2*, an interferon stimulated gene located in chromosome 19p13.2 [12]. The gene is 2.69 kb long and comprises of four functional exons [13, 14]. A previous study reported that *bst*-2 gene variants rs3217318, a 19-base-pair insertion/deletion polymorphism in the promoter region and rs10415893, a tag single-nucleotide polymorphism (SNP) in the 3′ untranslated region were associated with HIV-1 disease progression in a Spanish cohort [15]. A subsequent study showed that SNPs rs9576 and rs919266 were associated with disease outcome, with rs9576 marginally associated with protection against mother-to-child HIV-1 transmission in a Zambian cohort while SNP rs919266 was associated with slower progression to AIDS in Brazilian and Italian cohorts [16]. A different study showed that SNP rs919267 was associated with faster HIV-1 disease progression in an Indian cohort [17]. Collectively, these data suggest that *bst*-2 genetic variants may have a preventative or modifying role in HIV-1 infection or disease progression. However, the influence of *bst-*2 SNPs on endogenous BST-2 expression or other underlying mechanisms remain to be determined.

The HIV-1 accessory protein Vpu counteracts the restriction activity of BST-2 by targeting it for proteasomal and/or lysosomal degradation [12, 18]. Thus Vpu shields HIV-1 infected cells from immune clearance [19]. Studies have reported that treating HIV-1 infected cells with cytokines such as interferon alpha (IFN-α), IFN-β and interleukin 27 (IL-27) reversed the antagonistic effect of Vpu on BST-2 in a dose dependent manner [20, 21]. Specifically, these data showed that anti-HIV-1 neutralizing antibodies (NAbs) 3BNC117, PGT126 and PG9 are capable of mediating antibody-dependent cell-mediated cytotoxicity (ADCC) against HIV-1 infected T cells [20]. Moreover, BST-2 enhanced protective immune responses mediated by cells such as NK cells, CD4^+^ T cells and CD8^+^ T cells correlated with decreased infection levels [22]. A different study reported that high p24 specific IgG1 levels associated with antibody-mediated cellular phagocytosis (ADCP) responses [23]. Overall, the aforementioned studies suggest that higher expression of BST-2 may promote cell mediated antiviral activity in vivo and attenuate disease outcome.

However, the influence of *bst*-2 SNPs on expression levels, ADCC and/or ADCP activity has not been determined. We hypothesised that BST-2 SNPs may mediate BST-2 expression levels, ADCC and/or ADCP activity and disease outcome in a cohort of antiretroviral therapy naive, HIV-1 subtype C chronically infected individuals. Therefore, we investigated the effect of select *bst-2* SNPs rs919266, rs919267 and rs9576 on endogenous BST-2 expression levels, anti-HIV ADCC and/or ADCP activity and HIV-1 clinical outcomes.

## Results

### Association of endogenous BST-2 expression with HIV-1 status

**Fig. 1:**
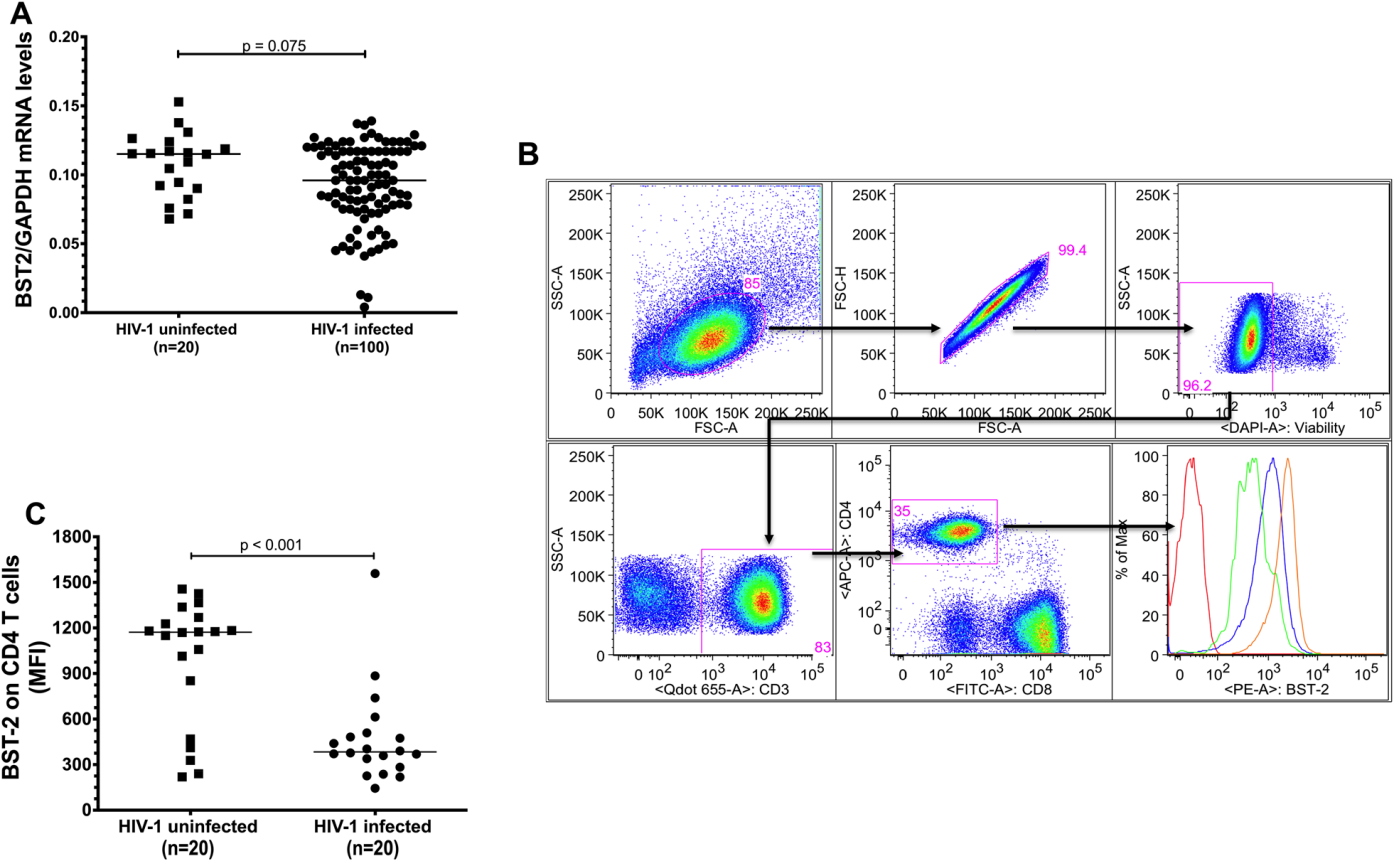
BST-2 expression levels in PBMCs and on CD4^+^ T cells from HIV-1 uninfected and infected participants. **A** BST-2 mRNA levels were compared between 20 HIV-1 uninfected (black *squares*) and 100 HIV-1 chronically infected participants (black circles) selected at baseline from the Sinikithemba chronic infection cohort. **B** A representative flow cytometry plots showing gating strategy for CD4^+^ T cell population from lymphocytes. Histograms show low (green), mid (blue) and high (orange) BST-2 expression intensity compared to the BST-2 FMO (red). **C** BST-2 surface staining on 20 HIV-1 uninfected (black squares) and 20 HIV-1 infected (black circles) participants. Horizontal lines within the clusters are depicting the median. Group comparisons of PBMCs and CD4^+^ T-cells were performed using the Mann–Whitney test

A previous study showed that BST-2 is upregulated during HIV-1 infection [24]. Thus, we hypothesized that BST-2 expression levels will be higher in HIV-1 subtype C chronically infected participants (Table 1). Our data show a trend towards lower BST-2 mRNA levels in PBMCs from HIV-1 infected compared to uninfected participants (p = 0.075), despite heterogeneous expression in both groups (Fig. 1A). Next, we investigated whether endogenous BST-2 mRNA expression levels in PBMCs translated to BST-2 protein expression levels on the surface of CD4^+^ T cells, the main target cells of HIV-1 infection in vivo. Samples from 20 HIV-1 uninfected and 20 randomly selected HIV-1 infected participants were analysed for BST-2 protein expression on CD4^+^ T cells using flow cytometry (Fig. 1B). CD4^+^ T cells from HIV-1 infected participants displayed significantly lower BST-2 protein expression as shown by lower median florescence intensity (MFI) compared to those from HIV-1 uninfected participants (p < 0.001) (Fig. 1C). While investigating Vpu expression among HIV-1 infected samples was outside the scope of this study, we could not analyse the cytokine levels due to plasma sample unavailability. Consistent with the recent report by Singh et al. [25], our data show that BST-2 expression is downregulated during HIV-1 infection.

**Table 1:** Demographic, clinical, and analytical characteristics of HIV-1 uninfected and infected participants

Characteristics	HIV-1 uninfected	HIV-1 infected	p-value
No. of patients (%)	20 (14.1)	123 (85.90)	0.0369
Age (yrs) mean ± SD (rage)	17.58 ± 1.99	32 ± 13.31	0.4011
Gender, male/female ratio	0/20	22/101	N/A
Median CD4 counts [IQR]	652 IQR [451.25–817.50]	480 IQR [329.75–670.00]	0.3668
Median Viral loads [IQR]	N/A	4.23 log_10_ IQR [2.97–5.03]	N/A

### Frequency of select *bst*-2 SNPs rs919267, rs9576 and rs919266 in HIV-1 positive and negative South Africans

Next we hypothesized that heterogenous BST-2 expression levels are at least partially mediated by its genetic variation. To test this hypothesis, select SNPs rs919267, rs9576 and rs919266 previously reported to have a preventative role during HIV-1 infection [16, 17] were genotyped in a total of 20 HIV-1 uninfected and 123 HIV-1 infected participants. SNP frequencies were analyzed and described for the HIV-1 infected participants only, since the number (n = 20) of HIV-1 uninfected participants was small due to sample availability. All polymorphisms analysed satisfied the Hardy–Weinberg equilibrium for allele frequencies (Table 2). The frequency of the wild type genotype (CC) for SNP rs919267 (rs919267CC) in HIV-1 infected participants was 41.5% while the heterogenous rs919267CT and homozygous rs919267TT mutants were 39.8% and 18.7%, respectively. The frequency of wild type genotype (CC) for SNP rs9576 (rs9576CC) was 79.5% whereas the heterozygous mutant rs9576CA was 20.5% among HIV-1 infected participants while there were no participants harbouring a homozygous rs9576AA mutant. SNP rs919266 was infrequent with the minor allele (rs919266T) frequency of 1.2% among HIV-1 infected participants and was therefore excluded from any further analysis.

**Table 2:** Frequency of BST-2 Polymorphism in HIV-1 subtype C chronically infected cohort and uninfected cohorts of black South Africans

SNP		Study population		HI vs HU	
		HIV infected (HI)	HIV uninfected (HU)	OR [95% CI]	p-value
MAF		n = 123 (%)	n = 20 (%)		
rs919266	A	3 (1.2)	2 (5.0)	N/A	N/A
rs919267	T	95 (38.6)	14 (65.0)	0.29 [10.30–9.72]	< 0.0001
rs9576	A	25 (11.0)	4 (10.0)	N/A	N/A
rs919266	G/G	120 (97.6)	18 (90.0)		
	G/A	3 (2.4)	2 (10.0)		
	AA	0 (0.0)	0 (0.0)		
rs919267	C/C	51 (41.5)	3 (15.0)		
	C/T	49 (39.8)	8 (40.0)		
	TT	23 (18.7)	9 (45.0)		
rs9576	C/C	97 (79.5)	16 (80.0)		
	C/A	25 (20.5)	4 (20.0)		
	AA	0 (0.0)	0 (0.0)		

### Association of *bst2* SNPs with BST-2 expression levels among HIV-1 uninfected and infected participants

**Fig. 2:**
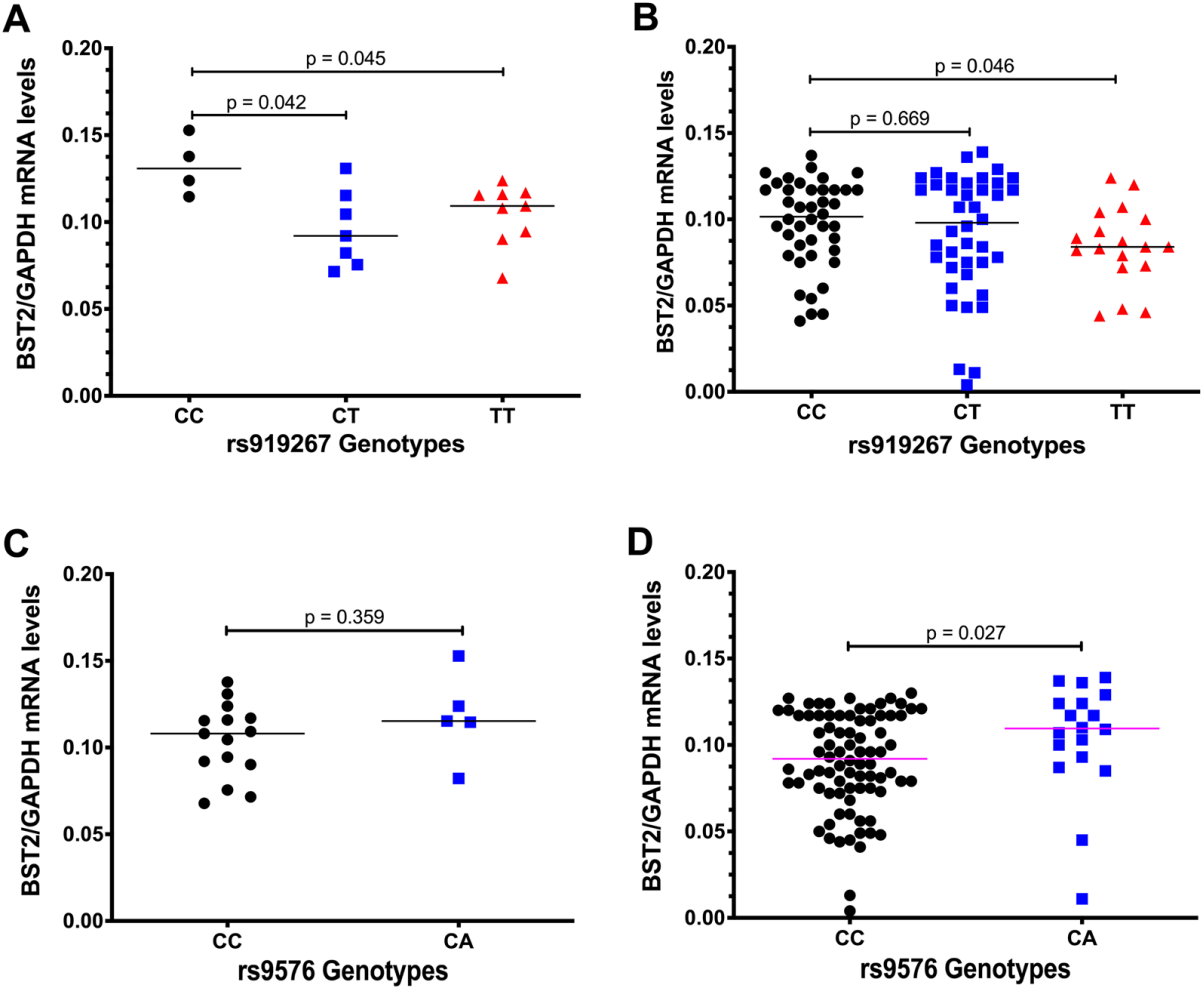
Association of *bst-2* genetic variation (rs919267 and rs9576) with BST-2 expression levels. **A** BST-2 mRNA expression levels in PBMCs obtained from HIV-1 uninfected participants with known genotypes, wildtype rs919267CC (black circles), heterozygous mutant rs919267CT (blue squares) and homozygous mutant rs919267TT (red tringles). **B** BST-2 mRNA expression levels in PBMCs obtained from HIV-1 infected participants with known genotypes, wildtype rs919267CC (black circles), heterozygous mutant rs919267CT (blue squares) and homozygous mutant rs919267TT (red tringles). **C** BST-2 mRNA expression levels in PBMCs obtained from HIV-1 uninfected participants with known genotypes, wildtype rs9576CC (black circles), heterozygous mutant rs9576CA (blue squares). **D** BST-2 mRNA expression levels in PBMCs obtained from HIV-1 infected participants with known genotypes, wildtype rs9576CC (black circles), heterozygous mutant rs9576CA (blue squares). Group comparisons of PBMCs were performed using the Mann–Whitney test

Next we explored whether heterogenous BST-2 expression was associated with *bst*-2 genetic variation for the polymorphic *bst*-2 SNPs rs919267 and rs9576. Remarkably, both the heterozygous mutant genotype rs919267CT (p = 0.042) and homozygous mutant rs919267TT (p = 0.045) were associated with significantly lower BST-2 mRNA median expression levels compared to the wild type genotype rs919267CC among HIV-1 uninfected participants (Fig. 2A). Our data further show that BST-2 mRNA expression levels differed across the rs919267 variants such that although similar expression was noted between the rs919267CT versus rs919267CC variants (p = 0.669), the rs919267TT homozygous mutant genotype was associated with significantly lower BST-2 mRNA median expression levels compared to rs919267CC among HIV-1 infected participants (p = 0.046) (Fig. 2B).

BST-2 mRNA median expression levels of the mutant rs9576CA genotype were not significantly different from the wild type rs9576CC genotype (p = 0.359) among the HIV-1 uninfected participants (Fig. 2C). In contrast, rs9576CA associated with significantly higher BST-2 mRNA median expression levels compared to rs9576CC (p = 0.027) among HIV-1 infected participants (Fig. 2D). Taken together, our data suggest that *bst*-2 SNPs rs919267 and rs9576 may regulate BST-2 expression levels following infection. However, it is also possible that *bst*-2 SNPs rs919267 and rs9576 could impact the sensitivity of the cell to infection and therefore affect viral loads. This could be due to the fact that *bst*-2 SNPs rs919267 and rs9576 can plausibly affect the BST-2 expression in the cells.

### Association of *bst2* SNPs with CD4 counts and viral loads

**Fig. 3:**
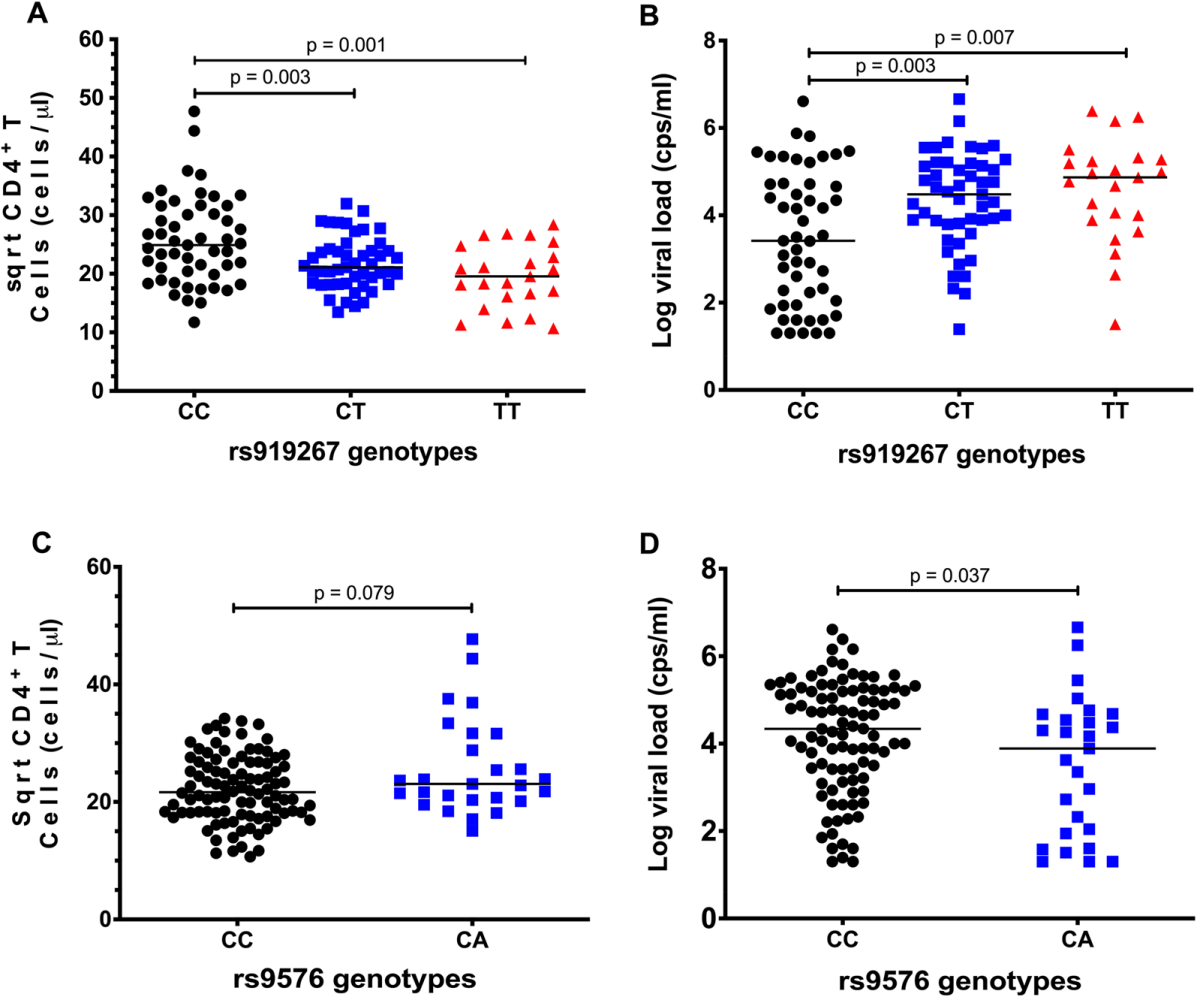
Association of SNP rs919267 and rs9576 with CD4 counts and viral loads in the Sinikithemba Chronic cohort. **A** Comparison of wildtype genotype rs919267 CC (black circles) with rs919267 CT (blue squares) and rs919267 TT (red triangle) in correlation with Square root of CD4^+^ T cell counts. **B** Comparison of wildtype genotype rs919267 CC (black circles) with rs919267 CT (blue squares) and rs919267 TT (red triangle) in correlation with viral loads. **C** Comparison of wildtype genotype rs9576 CC (black circles) with rs9576 CA (blue squares) in correlation with Square root of CD4^+^ T cell counts. **D** Comparison of wildtype genotype rs9576 CC (black circles) with rs9576 CA (blue squares) in correlation with viral loads. Group comparisons were performed using the Mann–Whitney test

A previous study reported that rs9576 was nominally associated with protection against infection during breastfeeding in a Zambian cohort [16] while rs919267 was associated with the risk of faster disease progression in an Indian cohort [17]. Therefore, we hypothesized that SNPs rs919267 and rs9576 may be associated with markers of disease progression such as viral loads and CD4 counts at baseline. Our data show that rs919267CT (p = 0.003) and rs919267TT (p = 0.001) were associated with significantly lower CD4 counts compared to rs919267CC, respectively (Fig. 3A). Conversely, rs919267CT (p = 0.003) and rs919267TT (p = 0.007) were associated with significantly higher viral loads compared to rs919267CC (Fig. 3B). On the other hand, rs9576CA (p = 0.079) was associated with a non-significant trend towards higher CD4 counts compared to rs9576CC (Fig. 3C). In contrast, rs9576CA (p = 0.037) was associated with significantly lower viral loads compared to rs9576CC (Fig. 3D). Taken together, our data suggest that *bst*-2 SNPs rs919267 and rs9576 mediate endogenous BST-2 expression and disease outcome.

### *bst*-2 SNPs rs919267 and rs9576 correlate gp120 IgG1 and IgG3 levels in HIV-1 infected participants

Previous studies reported that p24- but not gp120-specific antibody levels are a prognostic marker of disease progression in subtype B infection [26–28]. Consistently, the data from our group showed that p24-specific IgG3 responses are associated with poor viral control while p24-specific IgG1 responses may be a marker of viral control in HIV-1 subtype C infection [23]. However, the underlying mechanisms responsible for the lack of association of gp120-specific antibody responses with markers of disease progression remained to be determined. BST-2 is a restriction factor that tethers HIV-1 virions thus resulting in increased expression of viral envelope (gp120) on the surface of infected cells and killing of infected cells [1, 20]. Therefore, we hypothesized that endogenous BST-2 expression levels, regulated by *bst*-2 SNPs rs919267 and rs9576 may be correlated with gp120 IgG levels, ADCC and ADCP activity in plasma of HIV-1 infected individuals. To test this possibility, we investigated the association of rs919267 and rs9576 genotypes with titres of gp120 IgG subclasses, ADCC and ADCP activity of HIV-1 infected patients.

**Fig. 4:**
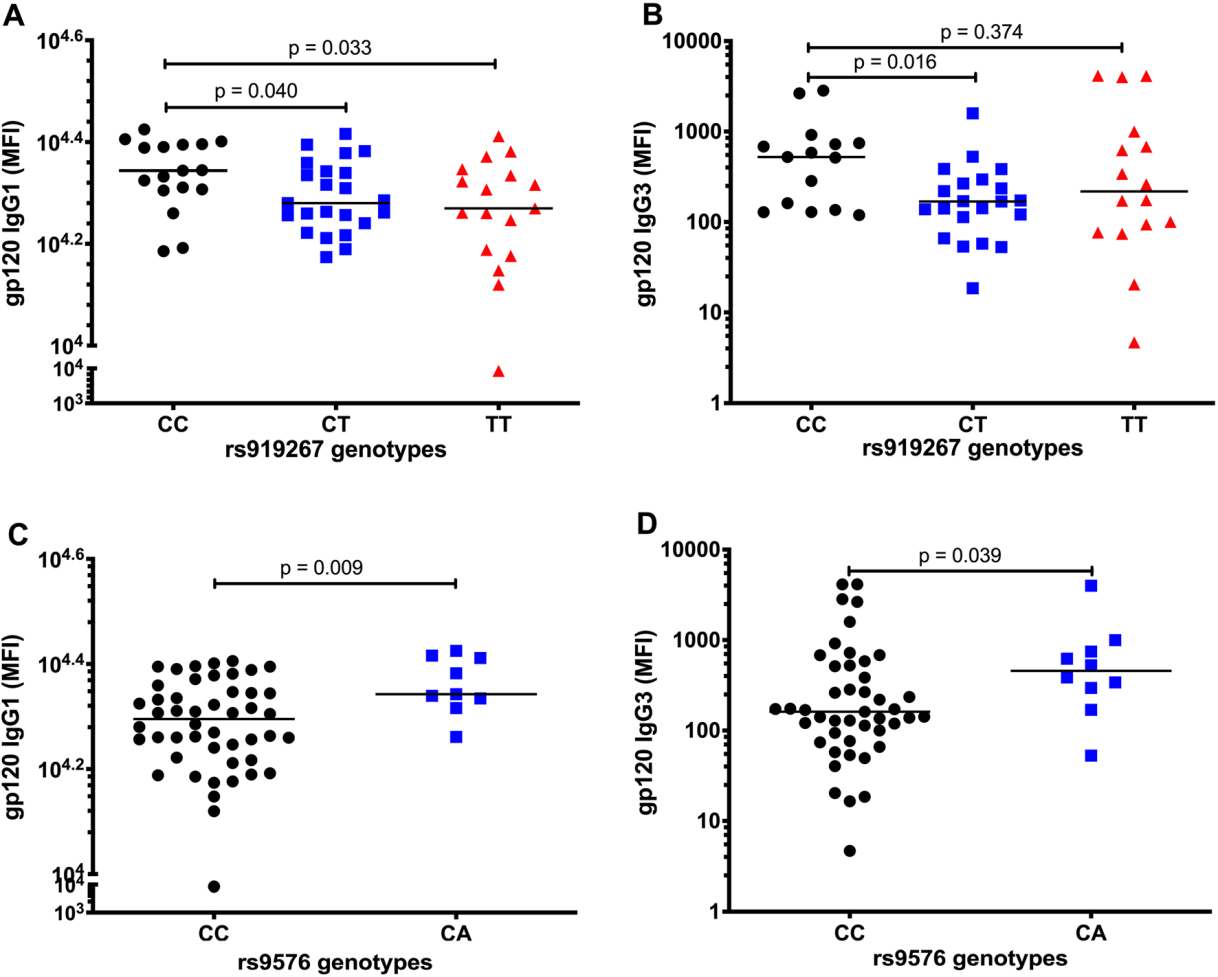
Association of SNP rs919267 and rs9576 with gp120-IgG1 and -IgG3 levels in the Sinikithemba chronic infection cohort. **A**, **B** Correlation of wildtype genotype rs919267CC (black circles) with rs919267CT (blue squares) and rs919267TT (red triangle) with gp120-IgG1 and -IgG3 MFI as percentage positive cells respectively. **C**, **D** Correlation of wildtype genotype rs9576**CC** (black circles) with rs9576**CA** (blue squares) with gp120-IgG1 and -IgG3 MFI as percentage positive cells respectively. Group comparisons were performed using the Mann–Whitney test

Interestingly, rs919267CT (p = 0.040) and rs919267TT (p = 0.033) were associated with significantly lower gp120 IgG1 levels compared to rs919267CC (Fig. 4A). In addition, rs919267CT was associated with significantly lower gp120 IgG3 levels (p = 0.016) while there was no difference in gp120 IgG3 levels for rs919267TT (p = 0.374) compared to rs919267CC (Fig. 4B). On the other hand, rs9576CA was associated with significantly higher gp120 IgG1 levels (p = 0.009) (Fig. 4C) and gp120 IgG3 levels (p = 0.039) compared to rs9576CC (Fig. 4D). However, rs919267 and rs9576 did not correlate with p24-IgG subclasses (Additional file 1), gp120 specific ADCP acitivity referred to as gp120 phagoscore and gp120 CD107α as a surrogate marker for degranulation or ADCC (Additional file 2). These data suggest that *bst*-2 SNPs rs919267 and rs9576 may mediate gp120-IgG1 and -IgG3 levels but not p24-IgG subclass levels, ADCC and ADCP activity.

## Discussion

BST-2 blocks the release of new HIV-1 virions from infected cells in vitro [1, 12]. In this study, we investigated the association of select *bst*-2 SNPs rs919266, rs919267 and rs9576 with endogenous BST-2 expression levels, HIV-1 disease outcome, ADCC and ADCP activity in two South African cohorts. Our findings from the current study show that HIV-1 chronic infection is associated with a trend towards lower BST-2 mRNA median expression in PBMCs from HIV-1 infected compared to uninfected participants. Consistently, we further found that median BST-2 surface protein expression on CD4^+^ T cells was significantly lower in HIV-1 chronically infected compared to uninfected participants. Although we could not perform Western blot analysis in the present study due to limited sample availability, we show here by flow cytometry that BST-2 expression on CD4^+^ T cells is lower in HIV infected compared to uninfected participants which is a similar trend observed for BST-2 mRNA expression in PBMCs. The data from the current study are consistent with the recent report by our group [25], which showed that lower BST-2 mRNA levels correlated with lower BST-2 protein expression levels as measured by Western blot. Therefore, it is likely that mRNA levels correlate with protein expression for the studied *bst*-2 SNPs rs919267 and rs9576 even though we were unable to directly prove this point. Although previous studies reported that HIV-1 accessory protein Vpu counteracts BST-2 on the surface of the infected cell by directing BST-2 for degradation [1, 12, 29], the effect of Vpu on BST-2 expression was outside the scope of this study. However, it is noteworthy that despite lower BST-2 median expression levels among HIV-1 infected compared to uninfected participants in the current study, BST-2 was heterogeneously expressed.

There are many factors that could contribute to differential immune gene expression in a population including immunity status, gender, age, environment and genetics in addition to virus production and IFN secretion (reviewed in [30]). In the present study we undertook to investigate the impact of select genetic variants, *bst*-2 SNPs rs919267, rs919266 and rs9576 on gene expression since previous studies reported that these SNPs are associated with disease outcome in different cohorts [16, 17]. However, the effect of these SNPs on BST-2 expression levels was not determined in the previous studies. Both SNPs rs919266 and rs919267 are located in the intronic region while rs9576 is an exonic SNP located in the 3’ untranslated region (UTR) of *bst*-2 gene. In the current study we show that rs919267CT and rs919267TT are associated with significantly lower BST-2 mRNA median expression levels compared to rs919267CC in PBMCs from HIV-1 uninfected participants. The heterozygous variant rs919267CT exhibited heterogenous BST-2 mRNA expression but similar expression levels to rs919267CC, whereas rs919267TT was associated with significantly lower median mRNA expression levels compared to rs919267CC in PBMCs from HIV-1 infected participants.

Although the mRNA expression levels were similar between rs9576CC and rs9576CA in PBMCs from HIV-1 negative participants, rs9576CA was associated with significantly higher BST-2 mRNA median expression levels compared to rs9596CC in HIV-1 positive participants suggesting protection against HIV-1 disease progression. Our data are consistent with a report showing that rs9576A allele was nominally associated with protection during breastfeeding in a cohort of Africans from Zambia [16]. It is well known that introns regulate gene expression but their mechanisms of action remain unclear [31, 32]. Although SNP rs919266A was previously reported to be associated with slower progression to AIDS [16], it was uncommon in this study population occurring only in 1.2% of HIV-1 infected and 5% of HIV-1 uninfected participants and therefore, it was excluded from further analysis. The data from the present study suggest that *bst*-2 SNPs rs919267 and rs9576 modulate BST-2 expression levels, which could correlate with disease outcome. Alternatively, *bst*-2 SNPs rs919267 and rs9576 may be associated with BST-2 expression in the cell which is consistent with a previous report showing that cells in which BST-2 expression was highly enhanced exhibited effective inhibition of HIV-1 production and replication even in the presence of viral antagonist Vpu against BST-2 [33]. Further research is necessary to determine, which of the two scenarios is mediated by *bst*-2 SNPs rs919267 and rs9576 or could possibly mediate both scenarios.

Next, we investigated the association of SNPs rs919267 and rs9576 with viral loads and CD4 counts. We found that rs919267CA and rs919267TT were also associated with lower CD4 counts but higher viral loads compared rs919267CC. These findings are in line with a previous study that was conducted in the Indian cohort, which reported that rs919267CT associated with higher risk to HIV-1 disease progression [17]. On the other hand rs9576CA associated with a trend towards higher CD4 counts and lower viral loads when compared to rs9576CC. These data are also consistent with the findings reported by Kamada et al*.* where rs9576CA was associated with protection in mother-to-child transmission in a Zambian cohort [16]. Collectively, our data suggest that increased BST-2 expression levels in vivo correlate with better disease outcome, reduced HIV-1 viral loads and higher CD4 counts.

Lastly, we investigated the mechanisms by which rs919267 and rs9576 mediated BST-2 expression modulate disease outcome. High levels of IgG1 and IgG3 antibodies are generally indicative of superior ability to provide a “first line of defense” against infections, including neutralization of viruses, increased ADCC and ADCP killing of virus-infected cells [34, 35]. HIV-1-specific IgG antibody responses have been shown to contribute to the control of HIV-1 infection [35]. Consistent with their association with lower BST-2 mRNA median expression, both rs919267CT and rs919267TT were associated with significantly lower gp120-IgG1 levels compared to rs919267CC. rs919267CT was further associated with significantly lower gp120-IgG3 levels while rs919267TT exhibited similar gp120-IgG3 levels compared to rs919267CC. On the other hand, consistent with its association with increased mRNA median expression levels, rs9576CA was associated with higher gp120-IgG1 and -IgG3 levels compared to rs9576CC. Alternatively, there is a possibility that the differences in gp120-IgG1 and gp120-IgG3 could be due to antigen structural diversity within a host. However, the genotypes of both SNPs rs919267 and rs9576 were not associated with the levels of p24-IgG subclasses, probably due to the fact that p24 is an internal viral protein. A previous report by Pharm et al. showed that increased ex vivo BST-2 levels results in efficient tethering of the virus on the surface of the infected cell, envelope recognition, binding of antibodies and ADCC killing by NK cells [20]. The data from the current study show that despite their association with BST-2 expression, viral loads, CD4 counts, gp120-IgG1 and -IgG3 levels, rs919267 and rs9576 are not correlated with gp120 phagoscore and gp120 CD107α meaning they do not modulate ADCP and ADCC activity. A negative correlation between viral loads and gp120-IgG1 and -IgG3 levels may indicate that the latter are contributing to viral control. However, in chronic infection, it is not only antibodies that may contribute a protective immune response and correlation may not always indicate causation and other unknown factors may contribute to this relationship.

## Conclusion

Taken together, the data from our study suggest that *bst*-2 SNPs rs919267 and rs9576 mediate BST-2 expression, disease outcome, gp120-IgG1 and -IgG3 levels in HIV-1 subtype C chronically infected people. However, rs919267 and rs9576 are not associated with p24 IgG levels, ADCC and ADCP activity suggesting that they use other mechanisms to control viral replication. Better understanding of the molecular mechanisms associated with BST-2 genetic variation to modulate HIV-1 disease outcome will be valuable for developing enhanced HIV-1 therapeutic and vaccine strategies. Our data are only suggestive and not confirmatory since we did not have enough samples to show that *bst*-2 SNPs rs919267 and rs9576 may regulate surface expression. Future studies where sample availability is not limited should investigate association with surface expression.

## Materials and methods

### Study participants and sample processing

The Sinikithemba cohort comprised of 450 antiretroviral naïve, HIV-1 subtype C chronically adults enrolled from McCord Hospital (Durban, South Africa) from August 2003 to 2008 and followed up longitudinally as previously described [36–38]. The time of infection for these participants is unknown. Sociodemographic characteristics, plasma viral load and CD4 count measurements were obtained at baseline. CD4 counts and viral loads were measured every 3 and 6 months from enrolment, respectively. Viral loads were determined using automated Cobas Amplicor HIV-1 Monitor test version 1.5 (Roche Diagnostics, Rotkreuz, Switzerland) and CD4^+^ T cells were enumerated using the Multitest kit CD4/CD3/CD8/CD45 on a FACSCalibur flow cytometer (Becton Dickinson). The Masibambisane cohort comprised of HIV negative women recruited from antenatal clinics in Durban [39]. Peripheral blood mononuclear cells (PBMCs) from participants were isolated by Ficoll-Histopaque (Sigma) density gradient centrifugation from blood within 6 h of phlebotomy and frozen in liquid nitrogen until use.

### Characterization of BST-2 mRNA expression levels in PBMCs from HIV-1 infected and uninfected participants

#### RNA extraction and cDNA synthesis

Cryopreserved PBMC samples from 20 HIV-1 uninfected and 123 HIV-1 infected participants from the Masibambisane and Sinikithemba cohorts respectively were available. Only 100 of 123 PMBC samples from HIV-1 infected participants were available for mRNA quantification.

Total RNA was extracted immediately after thawing and counting of PBMCs without stimulation. Total RNA was extracted from 2 × 10^6^ PBMCs using the RNeasy Mini kit (Qaigen, Hilden, Germany), according to the manufacturer’s protocol. Extracted RNA was DNase (ThemoFisher Scientific, Wilmington, USA) treated to digest away any carry over DNA, quantified before and after DNase treatment using the NanoDrop 2000 Spectrophotometer (ThemoFisher Scientific, Wilmington, USA) to make sure that contaminating DNA was eliminated. DNase treated RNA samples were used only if their OD_260_/OD_280_ ratio was 1.90 or greater. All RNA samples were DNase treated. Approximately, 1 µg of total RNA from each of the 20 HIV-1 uninfected and 100 HIV-1 infected (a total of 120) samples was reverse transcribed using the iScript cDNA synthesis kit as per the manufacturer’s instruction (BioRad Laboratories, Inc, Berkeley, CA).

#### Real-time PCR RNA quantitation

The levels of BST-2 mRNA were determined by a quantitative real-time PCR (qPCR) assay using SYBR Green chemistry in a LightCycler 480 (Roche). Each PCR reaction consisted of 3 mmol/µL MgCl_2_, the respective primers at 0.5 pmol/µL, 1 µL Fast Start SYBR Green I (Roche), 1 µg cDNA and water to make up the total reaction volume to 10 µL. The BST-2 cDNA was detected using the following primer set, BST-2 F: 5′-AGGTCCGTCCTGCTCGGCTT-3′ and BST-2 R: 5′-TCCAGAGGCCCTTCTCCGGC-3′ that are designed to specifically and uniquely amplify BST-2 (GenBank accession number NM_004335). Glyceraldehyde 3-phosphate dehydrogenase (GAPDH), (GenBank accession number NM_002046), determined to be the most suitable reference gene based on PCR efficiency in our laboratory [37] was used to correct for differences in the cDNA input. GAPDH cDNA was detected using the following primer set: GAPDH-F: 5′-AAGGTCGGAGTCAACGGATT-3′ and GAPDHR: 5′-CTCCTGGAAGATGGTGATGG-3′ as previously described [36]. The SYBR green qPCR was performed using the following program on the LightCycler 480: (1) preincubation: 95 °C for 5 min; (2) amplification: 45 cycles of 95 °C for 15 s, 60 °C for 15 s, 72 °C for 15 s; (3) melting curve: 95 °C for 5 s, 65 °C for 1 min, 97 °C for 0 s with a temperature transition rate of 0.11 °C/s [36]. To control for specificity of the amplification products, a melting curve analysis was performed. There was no amplification of non-specific products observed. The mRNA copy number was calculated from a standard curve, obtained by plotting known input concentrations of four different samples at log dilutions to the PCR cycle number (CP) at which the detected fluorescence intensity reaches a fixed value. The amplification efficiency of the PCR was determined by running log dilutions of standards. The slope of the standard curve was converted to the amplification efficiency.

### Measurement of BST-2 protein expression on CD4^+^ T cells from HIV-1 infected and uninfected participants by flow cytometry

Cryopreserved cells were rapidly thawed, washed twice in pre-warmed R10 medium (RPMI medium 1640 containing 10% gamma irradiated, heat inactivated fetal bovine serum (FBS) (Gibco, Life Technologies, Carlsbad, California, USA), 1% l-Glutamine, 1% penicillin/streptomycin and 1% HEPES buffer (1 molar) (Lonza, Basel, Basel-Stadt, Switzerland) at 500×*g* for 8 min at room temperature. Cells were rested in R10 medium at 37 °C for 2 h. Sample viability and cell counts were determined by trypan blue exclusion. One million cells were plated in 96-well plates and stained for 20 min with either LIVE/DEAD Fixable Blue or Near-IR Dead Cell Stain (Invitrogen, Carlsbad, California, USA), monoclonal antibodies CD3-brilliant violet 650 or brilliant violet 785 (clone OKT3, BioLegend, San Diego, California, USA), CD4-Allophycocyanin (clone SK3, BioLegend), CD8-Fluorescein (clone RPA-T8, BD Biosciences, Franklin Lakes, New Jersey, USA) and BST-2-Phycoerythrin (clone RS38E, BioLegend). Cells were washed with Dulbecco's phosphate-buffered saline, centrifuged at 850×*g* for 6 min and fixed with FIX & PERM Medium A (Invitrogen). Samples were acquired on a BD LSRFortessa. Routine instrument QC was performed using Cytometer Setup and Tracking beads (BD Biosciences). Compensation was calculated using the Anti-Mouse Ig, κ/Negative Control Compensation Particles Set (BD Biosciences). FlowJo software version 9 (TreeStar, Inc., Ashland, Oregon, USA) was used for sample analysis.

### Determination of the frequency of *bst*-2 SNPs rs919267, rs919266 and rs9576

The genotypes of select SNPs rs919267, rs919266 located in the intronic region and rs9576 located in 3’UTR were analysed in a total of 143 (20 HIV-1 uninfected and 123 HIV-1 infected) samples using TaqMan allelic discrimination assays as previously described [40]. Genomic DNA was isolated from stored buffy coats using the QIAamp DNA Blood Mini kit (Qiagen, Hilden, Germany) according to the manufacturer’s instructions. Briefly, 20 µL of QIAGEN Protease, 200 µL of AL Buffer were added to 200 µL of buffy coats, mixed by pulse-vortexing for 15 s and incubated at 56 °C for 10 min. Then 200 µL of ethanol (96–100%) was added to the sample and the mixture transferred to the QIAmp Mini column. Lastly, the QIAamp mini column was placed in a clean 1.5 mL microcentrifuge tube and DNA was eluted using 50 µL of AE Buffer. DNA concentration was standardised at 50 ng/μL with PCR grade water. A cocktail containing Taqman Genotyping master mix (Life Technologies, Carlsbad, California, USA) and probes for the *bst-2* gene (SNP ID: rs919266, rs919267 and rs9576, Applied Biosystems, Foster City, California, USA) was used to amplify target sequence in 50 ng genomic DNA by real time PCR in the LightCycler 480 (Roche, Basel, Switzerland) according to the manufacturer’s protocol.

### Association of *bst*-2 SNPs with IgG subclasses levels, ADCP and ADCC activity in antiretroviral-naïve HIV-1 subtype C chronic infection cohort

The IgG tires, ADCP and ADCC data were generated as previously described by Chung et al. [23]. Briefly, a 96-well plate was coated overnight at 4 °C with 150 ng of HIV gp120 consensus C or p24 subtype B/C protein per well to assay the gp120 IgG responses or p24 IgG responses respectively. 2% bovine serum albumin (BSA) blocked plates were used as antigen controls. The next day the plates were washed 6 times with PBS, 50 μL of plasma from HIV infected participants (diluted 1:100) was added to each well, and incubated at 37 °C for 2 h. HIV negative plasma samples or media alone were used as negative controls, while HIVIG (pooled HIV immunoglobulin G, NIH AIDS Reagents Program) was used as a positive control. The plates were washed and 5 × 10^4^ NK cells enriched via negative selection from healthy blood donors (RosetteSep, Stemcell Technologies,) were added to each well in the presence of Brefeldin A (BioLegend), Golgi stop, and anti-CD107α-PE-Cy5 (BD Biosciences). The plate was incubated for 5 h at 37 °C and 5% CO_2_. Following incubation, cells were stained with anti-CD3-AF700, anti-CD56-PE-Cy7, anti-CD16-APC-Cy7 (BD), fixed with Perm A, permeabilized using Perm B (Invitrogen), and stained with anti-IFNγ-APC and anti-MIP1β-PE (BD). The cells were then fixed with 2% paraformaldehyde and analysed by flow cytometry.

Statistical comparisons were examined by two-tailed Mann–Whitney *U* tests using GraphPad Prism software (GraphPad Inc., La Jolla, California, USA). A p-value < 0.05 was considered to be significant.

## Supplementary Information

**Additional file 1. **Association of rs919267 and rs9576 with p24 IgG1-IgG4 levels in the Sinikithemba Chronic cohort. **A**–**D:** Comparison of wildtype genotype rs919267CC (black circles) with rs919267CT (blue squares) and rs919267TT (red triangle) in correlation with p24 IgG MFI as percentage positive cells. **E–H:** Comparison of wildtype genotype rs9576CC (black circles) with rs9576CA (blue squares) in association with p24 IgG MFI as percentage positive cells.

**Additional file 2. ** Association of *bst-2* genetic variation (rs919267 and rs9576) with ADCP and ADCC activity. **A:** gp120 phagoschore as a surrogate marker of ADCP activity obtained from HIV-1 infected participants with known genotypes, wildtype rs919267CC (circles), heterozygous mutant rs919267CT (squares) and homozygous mutant rs919267TT (tringles). **B:** gp120 CD107α as a surrogate marker for degranulation obtained from HIV-1 infected participants with known genotypes, wildtype rs919267CC (circles), heterozygous mutant rs919267CT (squares) and homozygous mutant rs919267TT (tringles). **C:** gp120 phagoschore as a surrogate marker of ADCP activity obtained from HIV-1 infected participants with known genotypes, wildtype rs9576CC (circles), heterozygous mutant rs9567CT (squares). **D:** gp120 CD107α as a surrogate marker for degranulation obtained from HIV-1 infected participants with known genotypes, wildtype rs9576CC (circles), heterozygous mutant rs9576CA (squares). Group comparisons were performed using the Mann–Whitney test.

## Data Availability

All data generated or analysed during this study are included in this published article [and its supplementary information files]. The datasets used and/or analysed during the current study are available from the corresponding author on reasonable request.

## Abbreviations

ADCC: Antibody-dependent cell-mediated cytotoxicity
ADCP: Antibody-dependent cellular phagocytosis
BST-2: Bone marrow stromal antigen 2
HIV-1: Human immunodeficiency virus type 1
SNPs: Single nucleotides polymorphism

## References

[1] Neil SJ, Zang T, Bieniasz PD (2008). Tetherin inhibits retrovirus release and is antagonized by HIV-1 Vpu. Nature.

[2] Blasius AL, Giurisato E, Cella M, Schreiber RD, Shaw AS, Colonna M (2006). Bone marrow stromal cell antigen 2 is a specific marker of type I IFN-producing cells in the naive mouse, but a promiscuous cell surface antigen following IFN stimulation. J Immunol.

[3] Cao W, Bover L, Cho M, Wen X, Hanabuchi S, Bao M, Rosen DB, Wang YH, Shaw JL, Du Q (2009). Regulation of TLR7/9 responses in plasmacytoid dendritic cells by BST2 and ILT7 receptor interaction. J Exp Med.

[4] Loschko J, Heink S, Hackl D, Dudziak D, Reindl W, Korn T, Krug AB (2011). Antigen targeting to plasmacytoid dendritic cells via Siglec-H inhibits Th cell-dependent autoimmunity. J Immunol.

[5] Loschko J, Schlitzer A, Dudziak D, Drexler I, Sandholzer N, Bourquin C, Reindl W, Krug AB (2011). Antigen delivery to plasmacytoid dendritic cells via BST2 induces protective T cell-mediated immunity. J Immunol.

[6] Swiecki M, Wang Y, Gilfillan S, Lenschow DJ, Colonna M (2012). Cutting edge: paradoxical roles of BST2/tetherin in promoting type I IFN response and viral infection. J Immunol.

[7] Evans DT, Serra-Moreno R, Singh RK, Guatelli JC (2010). BST-2/tetherin: a new component of the innate immune response to enveloped viruses. Trends Microbiol.

[8] Mahauad-Fernandez WD, Okeoma CM (2016). The role of BST-2/Tetherin in host protection and disease manifestation. Immun Inflamm Dis.

[9] Liberatore RA, Bieniasz PD (2011). Tetherin is a key effector of the antiretroviral activity of type I interferon in vitro and in vivo. Proc Natl Acad Sci U S A.

[10] Halemano K, Barrett BS, Heilman KJ, Morrison TE, Santiago ML (2015). Requirement for Fc effector mechanisms in the APOBEC3/Rfv3-dependent neutralizing antibody response. J Virol.

[11] Jones PH, Mahauad-Fernandez WD, Madison MN, Okeoma CM (2013). BST-2/tetherin is overexpressed in mammary gland and tumor tissues in MMTV-induced mammary cancer. Virology.

[12] Van Damme N, Goff D, Katsura C, Jorgenson RL, Mitchell R, Johnson MC, Stephens EB, Guatelli J (2008). The interferon-induced protein BST-2 restricts HIV-1 release and is downregulated from the cell surface by the viral Vpu protein. Cell Host Microbe.

[13] Ozcan KA, Berndsen CE (2017). Bending of the BST-2 coiled-coil during viral budding. Proteins.

[14] Strauss JD, Hammonds JE, Yi H, Ding L, Spearman P, Wright ER (2016). Three-dimensional structural characterization of HIV-1 tethered to human cells. J Virol.

[15] Laplana M, Caruz A, Pineda JA, Puig T, Fibla J (2013). Association of BST-2 gene variants with HIV disease progression underscores the role of BST-2 in HIV type 1 infection. J Infect Dis.

[16] Kamada AJ, Bianco AM, Zupin L, Girardelli M, Matte MCC, De Medeiros RM, de Matos Almeida SE, Rocha MM, Segat L, Chies JAB (2016). Protective role of BST2 polymorphisms in mother-to-child transmission of HIV-1 and adult AIDS progression. J Acquir Immune Defic Syndr.

[17] Gupte DS, Patil A, Kumar CS, Pandey S, Achrekar SK, Paranjape RS, Jagtap DD (2020). Risk association of BST2 gene variants with disease progression in HIV-1 infected Indian cohort. Infect Genet Evol.

[18] Goffinet C, Allespach I, Homann S, Tervo HM, Habermann A, Rupp D, Oberbremer L, Kern C, Tibroni N, Welsch S (2009). HIV-1 antagonism of CD317 is species specific and involves Vpu-mediated proteasomal degradation of the restriction factor. Cell Host Microbe.

[19] Alvarez RA, Hamlin RE, Monroe A, Moldt B, Hotta MT, Caprio GR, Fierer DS, Simon V, Chen BK (2014). HIV-1 Vpu antagonism of tetherin inhibits antibody-dependent cellular cytotoxic responses by natural killer cells. J Virol.

[20] Pham TN, Lukhele S, Dallaire F, Perron G, Cohen ÉA (2016). Enhancing virion tethering by BST2 sensitizes productively and latently HIV-infected T cells to ADCC mediated by broadly neutralizing antibodies. Sci Rep.

[21] Richard J, Prévost J, Alsahafi N, Ding S, Finzi A (2018). Impact of HIV-1 envelope conformation on ADCC responses. Trends Microbiol.

[22] Li SX, Barrett BS, Guo K, Kassiotis G, Hasenkrug KJ, Dittmer U, Gibbert K, Santiago ML (2016). Tetherin/BST-2 promotes dendritic cell activation and function during acute retrovirus infection. Sci Rep.

[23] Chung AW, Makuba JM, Ndlovu B, Licht A, Robinson H, Ramlakhan Y, Ghebremichael M, Reddy T, Goulder P, Walker BD (2018). Viral control in chronic HIV-1 subtype C infection is associated with enrichment of p24 IgG1 with Fc effector activity. AIDS.

[24] Homann S, Smith D, Little S, Richman D, Guatelli J (2011). Upregulation of BST-2/tetherin by HIV infection in vivo. J Virol.

[25] Singh R, Ramsuran V, Naranbhai V, Yende-Zuma N, Garrett N, Mlisana K, Dong KL, Walker BD, Abdool Karim SS, Carrington M (2021). Epigenetic regulation of BST-2 expression Levels and the effect on HIV-1 pathogenesis. Front Immunol.

[26] Farzadegan H, Henrard DR, Kleeberger CA, Schrager L, Kirby AJ, Saah AJ, Rinaldo CR, O'Gorman M, Detels R, Taylor E (1996). Virologic and serologic markers of rapid progression to AIDS after HIV-1 seroconversion. J Acquir Immune Defic Syndr Hum Retrovirol.

[27] French MA, Abudulai LN, Fernandez S (2013). Isotype Diversification of IgG Antibodies to HIV Gag Proteins as a Therapeutic Vaccination Strategy for HIV Infection. Vaccines (Basel).

[28] Zwart G, van der Hoek L, Valk M, Cornelissen MT, Baan E, Dekker J, Koot M, Kuiken CL, Goudsmit J (1994). Antibody responses to HIV-1 envelope and gag epitopes in HIV-1 seroconverters with rapid versus slow disease progression. Virology.

[29] Iwabu Y, Fujita H, Kinomoto M, Kaneko K, Ishizaka Y, Tanaka Y, Sata T, Tokunaga K (2009). HIV-1 accessory protein Vpu internalizes cell-surface BST-2/tetherin through transmembrane interactions leading to lysosomes. J Biol Chem.

[30] Aguirre-Gamboa R, Joosten I, Urbano PC, van der Molen RG, van Rijssen E, van Cranenbroek B, Oosting M, Smeekens S, Jaeger M, Zorro M (2016). Differential effects of environmental and genetic factors on T and B cell immune traits. Cell Rep.

[31] Kalsotra A, Cooper TA (2011). Functional consequences of developmentally regulated alternative splicing. Nat Rev Genet.

[32] Yang YF, Zhu T, Niu DK (2013). Association of intron loss with high mutation rate in Arabidopsis: implications for genome size evolution. Genome Biol Evol.

[33] Zhang Y, Ozono S, Yao W, Tobiume M, Yamaoka S, Kishigami S, Fujita H, Tokunaga K (2019). CRISPR-mediated activation of endogenous BST-2/tetherin expression inhibits wild-type HIV-1 production. Sci Rep.

[34] Smith FL, Baumgarth N (2019). B-1 cell responses to infections. Curr Opin Immunol.

[35] French MA, Tjiam MC, Abudulai LN, Fernandez S (2017). Antiviral functions of human immunodeficiency virus type 1 (HIV-1)-specific IgG antibodies: effects of antiretroviral therapy and implications for therapeutic HIV-1 vaccine design. Front Immunol.

[36] Madlala P, Singh R, An P, Werner L, Mlisana K, Karim SSA, Winkler CA, Ndung’u T (2016). Association of polymorphisms in the regulatory region of the cyclophilin A gene (PPIA) with gene expression and HIV/AIDS disease progression. J Acquir Immune Defic Syndr.

[37] Madlala P, Gijsbers R, Christ F, Hombrouck A, Werner L, Mlisana K, An P, Karim SSA, Winkler CA, Debyser Z (2011). Association of polymorphisms in the LEDGF/p75 gene (PSIP1) with susceptibility to HIV-1 infection and disease progression. AIDS.

[38] Brumme Z, Wang B, Nair K, Brumme C, de Pierres C, Reddy S, Julg B, Moodley E, Thobakgale C, Lu Z (2009). Impact of select immunologic and virologic biomarkers on CD4 cell count decrease in patients with chronic HIV-1 subtype C infection: results from Sinikithemba Cohort, Durban, South Africa. Clin Infect Dis.

[39] Sharp CP, Gregory WF, Hattingh L, Malik A, Adland E, Daniels S, van Zyl A, Carlson JM, Wareing S, Ogwu A (2017). PARV4 prevalence, phylogeny, immunology and coinfection with HIV, HBV and HCV in a multicentre African cohort. Wellcome Open Res.

[40] Julg B, Reddy S, van der Stok M, Kulkarni S, Qi Y, Bass S, Gold B, Nalls MA, Nelson GW, Walker BD (2009). Lack of Duffy antigen receptor for chemokines: no influence on HIV disease progression in an African treatment-naive population. Cell Host Microbe.

