# Supplementary figures and images for: Bone marrow stromal antigen 2 (BST-2) genetic variants influence expression levels and disease outcome in HIV-1 chronically infected patients
Source: Retrovirology. 2022 Jan 26;19:3. doi: 10.1186/s12977-022-00588-2 (PMC8793201; doi:10.1186/s12977-022-00588-2)

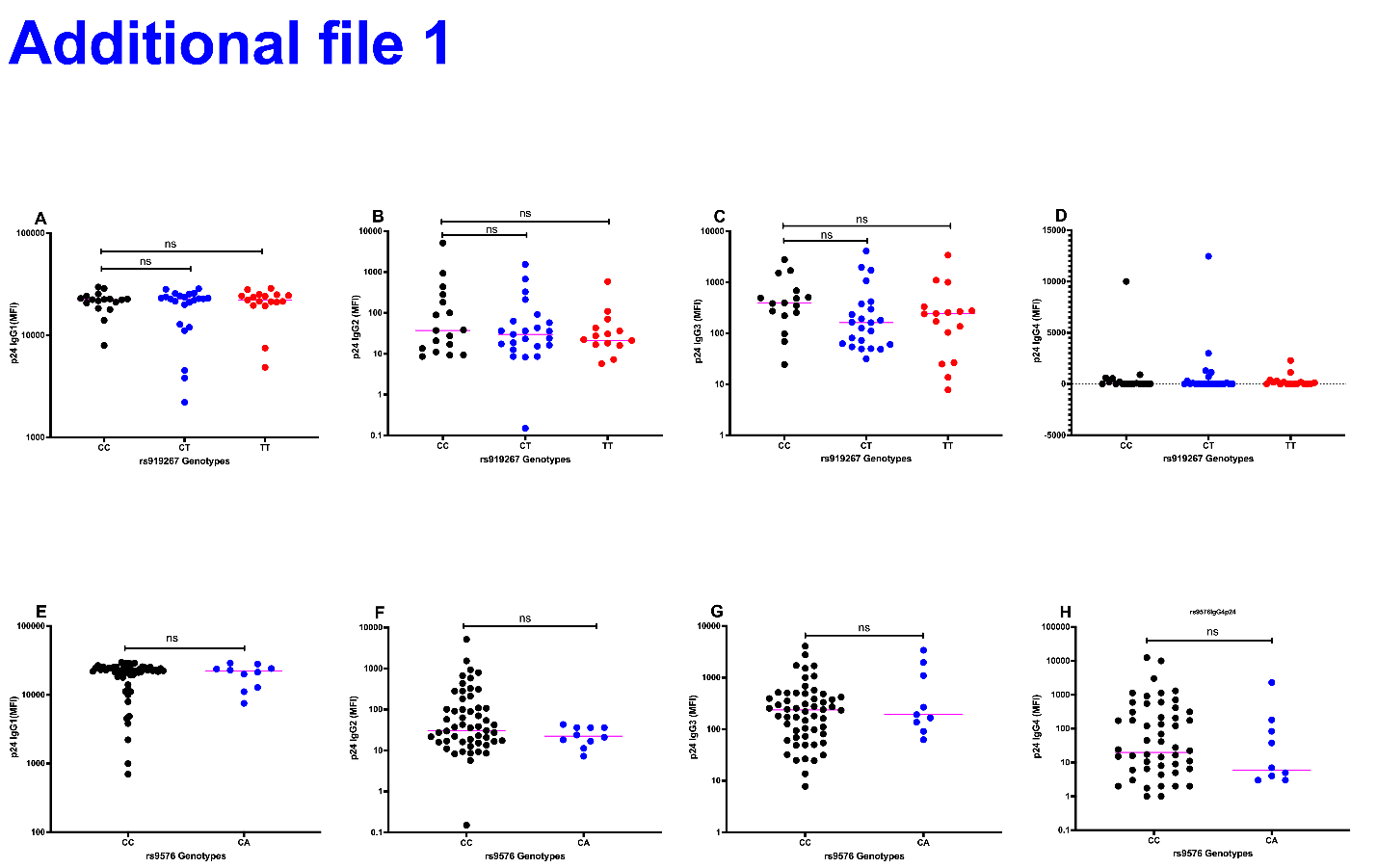

Supplement: Supplementary file 1 — Additional file 1. Association of rs919267 and rs9576 with p24 IgG1-IgG4 levels in the Sinikithemba Chronic cohort. A–D: Comparison of wildtype genotype rs919267CC (black circles) with rs919267CT (blue squares) and rs919267TT (red triangle) in correlation with p24 IgG MFI as percentage positive cells. E–H: Comparison of wildtype genotype rs9576CC (black circles) with rs9576CA (blue squares) in association with p24 IgG MFI as percentage positive cells. [file 12977_2022_588_MOESM1_ESM.docx]

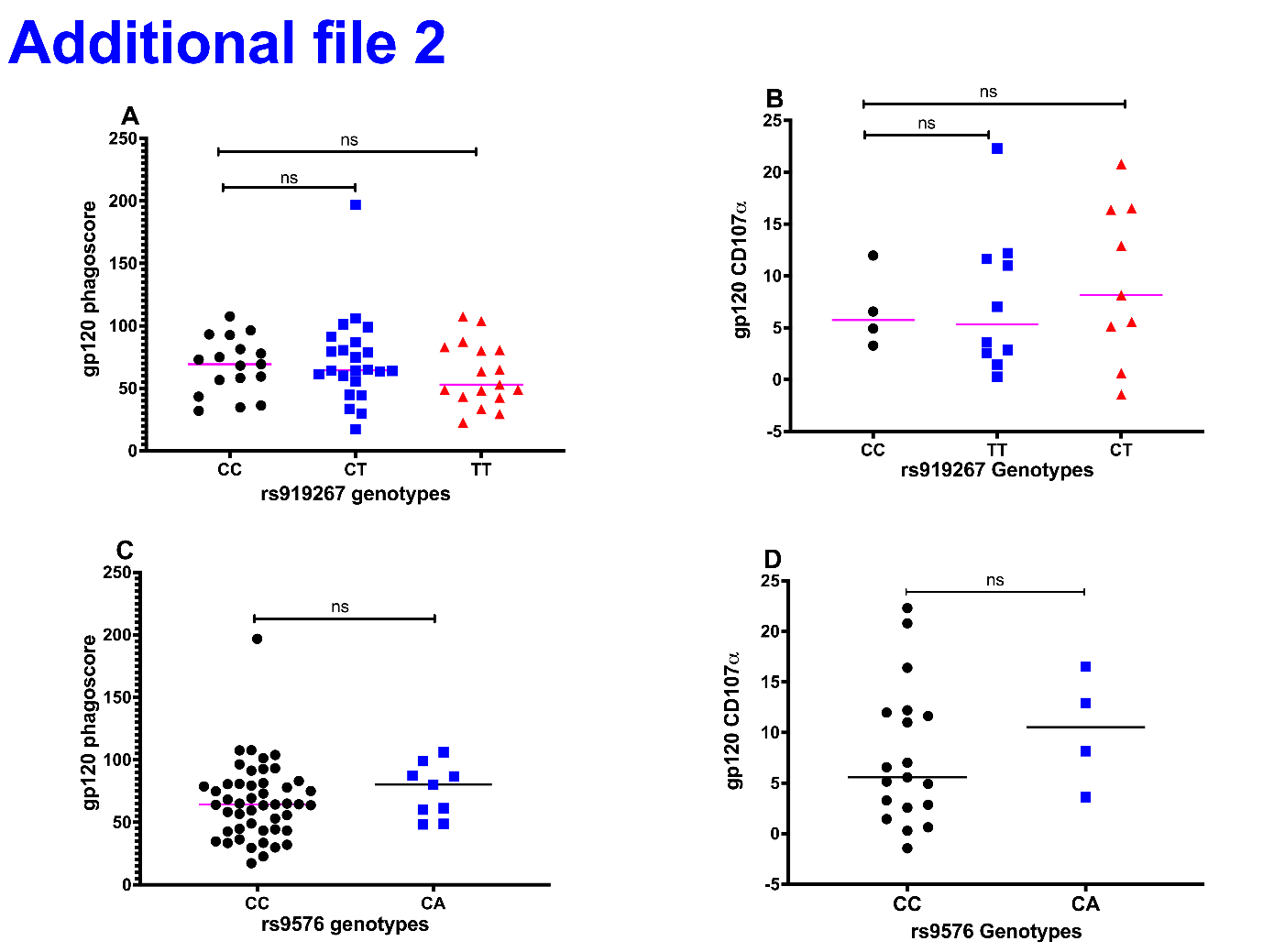

Supplement: Supplementary file 2 — Additional file 2. Association of bst-2 genetic variation (rs919267 and rs9576) with ADCP and ADCC activity. A: gp120 phagoschore as a surrogate marker of ADCP activity obtained from HIV-1 infected participants with known genotypes, wildtype rs919267CC (circles), heterozygous mutant rs919267CT (squares) and homozygous mutant rs919267TT (tringles). B: gp120 CD107α as a surrogate marker for degranulation obtained from HIV-1 infected participants with known genotypes, wildtype rs919267CC (circles), heterozygous mutant rs919267CT (squares) and homozygous mutant rs919267TT (tringles). C: gp120 phagoschore as a surrogate marker of ADCP activity obtained from HIV-1 infected participants with known genotypes, wildtype rs9576CC (circles), heterozygous mutant rs9567CT (squares). D: gp120 CD107α as a surrogate marker for degranulation obtained from HIV-1 infected participants with known genotypes, wildtype rs9576CC (circles), heterozygous mutant rs9576CA (squares). Group comparisons were performed using the Mann–Whitney test. [file 12977_2022_588_MOESM2_ESM.docx]
